# Adams–Oliver Syndrome: A Rare Congenital Disorder

**DOI:** 10.7759/cureus.23297

**Published:** 2022-03-18

**Authors:** Sumara Rashid, Saleha Azeem, Samiha Riaz

**Affiliations:** 1 Dermatology, Fatima Memorial College of Medicine and Dentistry, Lahore, PAK; 2 Dermatology, King Edward Medical University, Lahore, PAK

**Keywords:** adams–oliver syndrome, cutaneous lesions, hypoplastic phalanges, hypertrophic labia minora, aplasia cutis congenita

## Abstract

We present a case of a two-day-old Asian female infant with typical symptoms of Adams-Oliver syndrome (AOS): two cutaneous lesions including aplasia cutis congenita (ACC) and hypoplastic phalanges. The lesion on the abdomen is a relatively rare finding of the syndrome. Skin and skull bone were absent in the anterior fontanelle region, and hypertrophic labia minora was observed. The patient was put on regular follow-up.

## Introduction

Adams-Oliver syndrome (AOS), a rare congenital disorder, is characterized by congenital scalp defects (aplasia cutis congenita (ACC)) along with defects of the terminal transverse limbs that may vary in severity [1]. This disorder was described for the first time in 1945 by Adams and Oliver [2], and since then, several similar cases have been reported. Although autosomal dominant (AD) inheritance was thought to be the probable mode of inheritance initially in 1945, subsequent case reports suggest autosomal recessive (AR) inheritance for this syndrome. Having a family history of the syndrome and being born to parents that are closely related by blood are both genetic risk factors for AOS. Cutis marmorata telangiectatica congenita, an added defect, has been associated with 12% of cases of AOS [3]. Congenital defects and vascular heart anomalies may also be present [4]. The most common limb anomalies are hypoplastic or absent distal phalanges, but defects range from hypoplastic nails to completely absent hands or lower legs [5]. It is believed that normal lifespan is not affected if the syndrome does not involve any major organs [6]. The involvement of internal organs, including the central nervous system, cardiopulmonary system, and gastrointestinal tract, is usually lethal [5].

AOS is a rare disease and even rarer with lesions on the abdomen, which is why this case is being presented [7]. Reporting this case, especially with a unique finding, will contribute to the limited relevant literature that exists and that will lead to an overall better understanding of the syndrome.

## Case presentation

**Figure 1:**
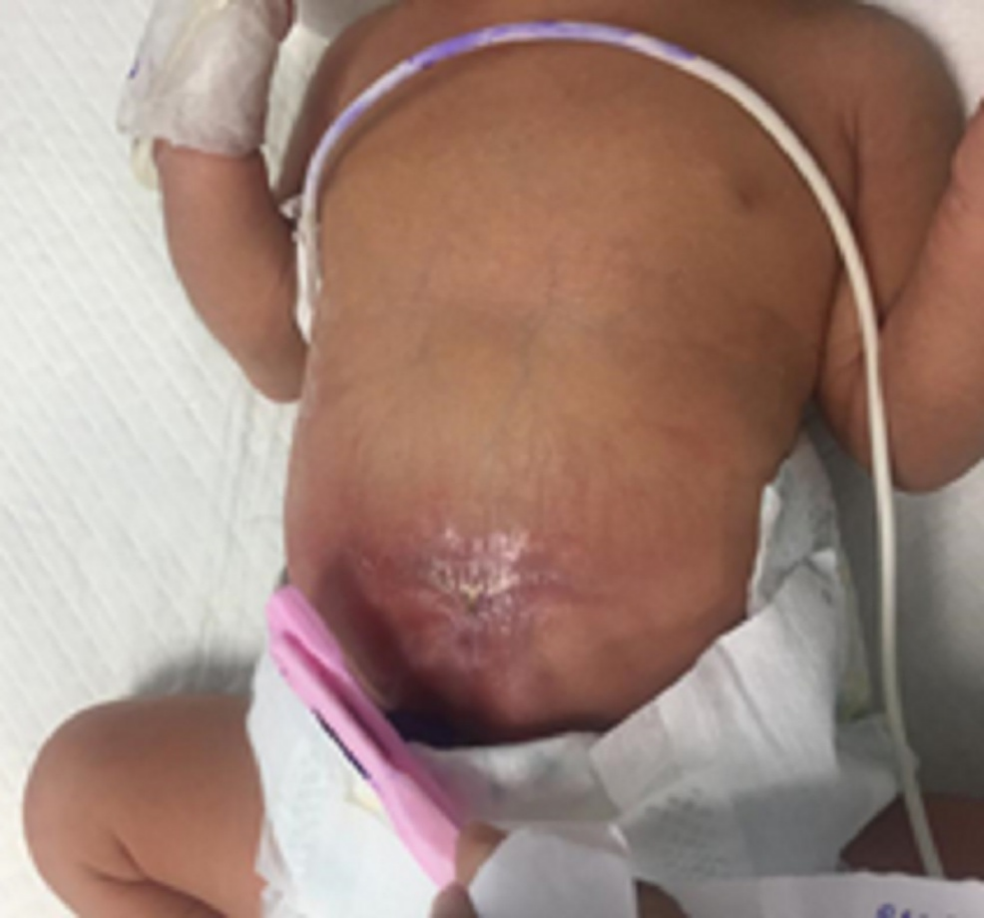
Cutaneous lesion above the umbilicus

**Figure 2:**
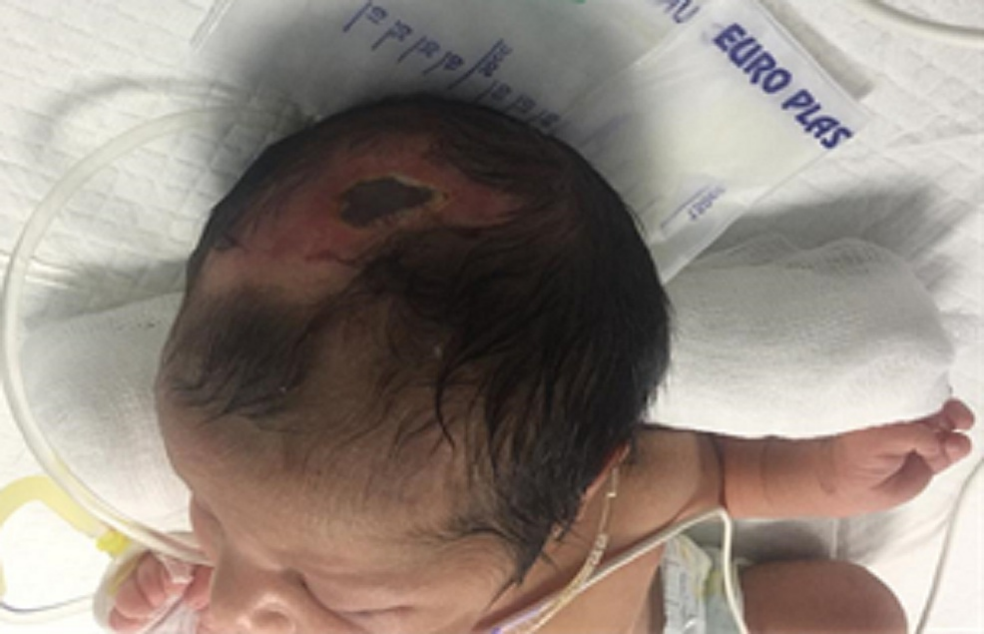
Cutaneous lesion on the central scalp

A two-day-old Pakistani female infant weighing 4.6 pounds at birth was born after a full-term pregnancy through C-section delivery. She was the second of two siblings and the daughter of consanguineous healthy parents. She was referred to the Dermatology Department at Fatima Memorial Hospital, Lahore, Pakistan, for the evaluation of two cutaneous lesions on the central line of her abdomen above the umbilicus (Figure 1) and on the central scalp (Figure 2). A focal defect of scarred atrophic plaque with thin skin was present in the midline of the vertex area involving the sagittal suture. The lesion was approximately 4 × 3 cm in the region anterior to the occipital fontanelle. The scarred plaque had a complete absence of hair and a central hemorrhagic necrotic crust. A small ulcerated area was observed in the immediate surroundings of the crust. The abdominal skin had a puckered, shiny scar on the central lower abdomen surrounding the umbilicus. It was a 3 × 3 cm erythematous area with a band-like small hypertrophic scar in its center, but there was no ulceration. Normal cutaneous blood vessels were visible in the surrounding area.

The mother denied any toxic exposures or any serious illness during pregnancy and any family history of the syndrome.

**Figure 3:**
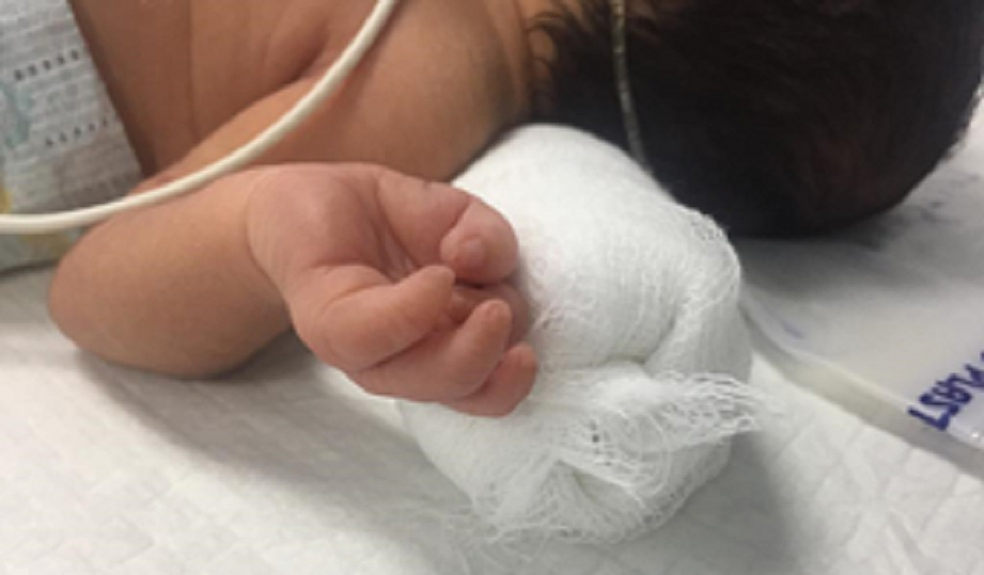
Hypoplastic phalanges of hands

**Figure 4:**
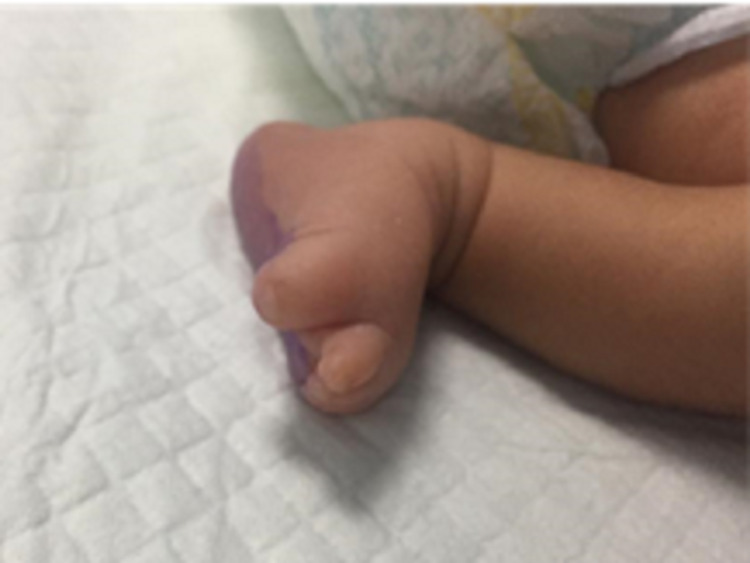
Hypoplastic phalanges of feet

Due to the presence of hypoplastic phalanges, both in digits and toes, the child was scheduled to undergo a complete musculoskeletal examination, X-ray of hands and feet, an abdominal ultrasound, an echocardiogram, and CT scan of the skull in the Department of Pediatrics (Figures 3, 4). Upon physical examination of the patient, it was revealed that the skin and parts of both parietal bones making the sagittal suture were absent in the occipital fontanelle region. Additionally, hypertrophic labia minora was observed. There were no other noticeable symptoms, and the child was healthy. Generalized cutis marmorata was not observed although it is associated with some cases of AOS. Moro reflex was intact. Suckling after birth and ophthalmological consultation were normal. The baby was lying in the midline position with a symmetrical body. Normal limb movements were present with normal tendon reflexes. The facial response to touch was of normal category. The baby was kept in a newborn nursery for observation and cardiac monitoring. Further imaging and other investigations were planned, and a topical antibacterial was prescribed for the scalp hemorrhagic ulcerated lesion. The parents were genetically counseled. Informed consent for the use of pictures and disease details was obtained from the parents.

## Discussion

Adams-Oliver syndrome has an approximate incidence of one in 225,000 births [4]. It was first picked up and reported by Adams and Oliver in 1945. They identified eight cases in one family [2]. Since then, cases of Adams-Oliver syndrome have been reported worldwide with various symptoms.

Cases with autosomal dominant (AD), sporadic, and autosomal recessive (AR) genetics have been identified [4]. The AD and AR AOS-related genes are ARHGAP31, DLL4, NOTCH1, or RBPJ and DOCK6 or EOGT, respectively [8]. Genetic predisposition is a speculated factor in the cases identified by Adams and Oliver. The absence of family history suggests this case to be most likely a sporadic one. However, it does not rule out its genetic inheritance. This is because parents that are closely related by blood have a greater chance of carrying the same abnormal gene as opposed to parents who are not. Therefore, a consanguineous marriage, such as the one in our case, increases the chance of AOS [8].

Terminal transverse limb defects, aplasia cutis congenita (ACC), and positive family history are considered as major criteria for the diagnosis of AOS [4,9]. Cutis marmorata, congenital heart defects, and vascular anomaly are considered minor criteria for the diagnosis of the syndrome. To label the diagnosis, two major criteria or one major and one minor are taken as adequate evidence [4].

Disproportionate limb deformities are the most commonly seen finding in AOS [4], especially involving the lower limbs [3], and may include hypoplastic fingernails or toenails, syndactyly, polydactyly, or brachydactyly. Oligodactyly, the complete absence of a finger, toe, limb, or hand, may be observed in severe cases [5]. Phenotypically, the appearance of the limbs can show varying severity from normal appearance (as in obligate AOS carriers) to the total absence of hand or foot.

The causes for aplasia cutis congenita may be teratogenic factors (physical, metabolic, infectious, chemical, or maternal health factors), fetal exposure to drugs such as cocaine and alcohol, or intrauterine infections [10]. No such history was seen in this case. The only significant part for the entire duration of the pregnancy was the suggestion of a C-section due to fetal distress exhibited as tachycardia at term. Fetal distress was not noted at any other stage of pregnancy as indicated by fetal Doppler studies. The lesions, however, were formed during the beginning of intrauterine life as indicated by the healed scar tissue on birth. Therefore, fetal distress cannot be a potential cause.

Although the typical site for ACC is scalp vertex, less common sites such as the parietal scalp, trunk (torso), and limbs can also be involved [11]. On the abdomen, the linear band of hypertrophic scar is confined to a specific area in the center. This is not expected to lead to abdominal constriction during the growth of the abdominal wall and musculature as might have happened in case the scar had extended to cover a greater area. A broad spectrum of intracranial abnormalities has been documented in AOS patients that include encephalocele, microcephaly, hypoplasia of the left arteria cerebri, medial and right spastic hemiplegia, cortical dysplasia, pachygyria, hypoplastic corpus callosum, parenchymal calcifications, abnormal cerebral vasculature, ventriculomegaly, and dysplasia of the cerebral cortex [3]. These could result in secondary symptoms such as epilepsy and mental retardation. Furthermore, in skin lesions on the scalp associated with AOS, underlying dilated blood vessels may bleed and lead to hemorrhage [8]. In our case, a necrotic hemorrhagic crust was seen in the center of the otherwise healed scalp lesion.

Labial abnormalities, as seen in our patient, have not been reported before. There is currently no biological reason, but it may be associated with a wide clinical spectrum of AOS. The cardiovascular system can also be affected in the form of obstructive defects in the left heart, valvular anomalies, pulmonary vascular malformation, and pulmonary hypertension. Other clinical abnormalities that can be seen in such patients include cutis marmorata telangiectasia congenita, gastrointestinal and hepatic malformations, accessory nipples, microphthalmia, hereditary hemorrhagic telangiectasia, and cleft lip [3]. Our patient did not show any of these findings as evident by examination and investigations.

Differential diagnoses can include epidermolysis bullosa, herpes simplex infection, and focal dermal hypoplasia (Goltz syndrome). The absence of bullous eruptions and the absence of the involvement of sites of friction sites rule out epidermolysis bullosa. Herpes simplex, although often found on the scalp, is not associated with limb or abdomen abnormalities. Focal dermal hypoplasia is a multisystem disorder involving the hair, teeth, glands, and eyes. Although the teeth of a newborn cannot be assessed, hair and eyes can, and they were normal. CT scan and MRI did not show any abnormalities in internal organs [12]. Aplasia cutis congenita could be a part of even rarer syndromes.

Although there is no lethal internal organ involvement, meningitis and infections can be potential complications, so a regular follow-up is essential. Genetic prenatal testing is recommended for future pregnancies, but it may not be easily accessible in Pakistan. Parents should be genetically counseled as to the gene for AOS has a 50% (AD inheritance) or a 25% (AR inheritance) chance of being inherited. During follow-up, a multidisciplinary approach may be required to take care of the associated limb abnormalities, e.g., plastic surgery for the hypoplastic limb defects or cutis aplasia. Vaginal surgeries for normal structural restoration may be considered after puberty. Regular follow-up, growth charts, and close monitoring by a pediatrician should be done. The quality of life may be compromised due to cosmetic disabilities, but in terms of life expectancy, the patient is expected to live a normal life provided that the necessary milestones are achieved and the skull bone grows normally without any serious comorbidity.

## Conclusions

AOS is a rare multisystem disorder that affects the quality of life and can be lethal if internal organs are involved. We present a case of AOS with two cutaneous lesions, hypoplastic phalanges, and hypertrophic labia minora. A regular follow-up, in this case, is required with immediate reporting of infection and disease. Any complication should be dealt with immediately and accordingly.
